# HIV-1 Vpr Induces Interferon-Stimulated Genes in Human Monocyte-Derived Macrophages

**DOI:** 10.1371/journal.pone.0106418

**Published:** 2014-08-29

**Authors:** Muhammad Atif Zahoor, Guangai Xue, Hirotaka Sato, Tomoyuki Murakami, Shin-nosuke Takeshima, Yoko Aida

**Affiliations:** 1 Viral Infectious Diseases Unit, RIKEN, Wako, Saitama, Japan; 2 Japanese Foundation of AIDS Prevention, Tokyo, Japan; Temple University School of Medicine, United States of America

## Abstract

Macrophages act as reservoirs of human immunodeficiency virus type 1 (HIV-1) and play an important role in its transmission to other cells. HIV-1 Vpr is a multi-functional protein involved in HIV-1 replication and pathogenesis; however, its exact role in HIV-1-infected human macrophages remains poorly understood. In this study, we used a microarray approach to explore the effects of HIV-1 Vpr on the transcriptional profile of human monocyte-derived macrophages (MDMs). More than 500 genes, mainly those involved in the innate immune response, the type I interferon pathway, cytokine production, and signal transduction, were differentially regulated (fold change >2.0) after infection with a recombinant adenovirus expressing HIV-1 Vpr protein. The differential expression profiles of select interferon-stimulated genes (ISGs) and genes involved in the innate immune response, including *STAT1, IRF7, MX1, MX2, ISG15, ISG20, IFIT1, IFIT2, IFIT3, IFI27, IFI44L, APOBEC3A, DDX58* (RIG-I), *TNFSF10* (TRAIL), *and RSAD2* (viperin) were confirmed by real-time quantitative PCR and were consistent with the microarray data. In addition, at the post-translational level, HIV-1 Vpr induced the phosphorylation of STAT1 at tyrosine 701 in human MDMs. These results demonstrate that HIV-1 Vpr leads to the induction of ISGs and expand the current understanding of the function of Vpr and its role in HIV-1 immune pathogenesis.

## Introduction

Antigen-presenting cells (APCs) are critical for both innate and adaptive immunity. Professional APCs such as macrophages play an integral role in the immune pathogenesis of the human immunodeficiency virus type 1 (HIV-1) [1]. HIV-1 is a member of the lentivirus family and is the etiologic agent of acquired immunodeficiency syndrome (AIDS). It interacts with host cells through multiple signaling pathways to establish the disease [2]. The infection involves complex mechanisms through which HIV-1 overcomes the host immune responses and causes reprogramming of the host transcriptome and proteome [3]–[5].

Vpr, an accessory gene product of HIV-1, is a protein of 96 amino acids and has a predicted molecular weight of 15 kDa that is relatively conserved in HIV-1 and simian immunodeficiency virus (SIV) [6]. Vpr is a pleiotropic protein that is involved in diverse functions including cell-cycle arrest at the G2/M phase [7], apoptosis [7]–[9], nuclear import of the pre-integration complex [10]–[14], transcriptional activation [15], and splicing [16], [17]. Vpr performs these functions through interactions with various host cellular factors such as DCAF1, SAP145, p300, and importin-α [8], [10], [11], .

A striking feature of Vpr is its unique potential to promote viral productivity in monocytes/macrophages and in a small population of CD4^+^ T-cells [22]–[26]. Although Vpr is thought to play an important role in HIV-1-infected human macrophages [1], [3], [6], [11], [21], [23], little is known about how it disrupts the expression profile of host cellular genes. In this study, we analyzed the effect of Vpr on the expression profiles of host cellular genes in human monocyte-derived macrophages (MDMs), with the idea that such an analysis would provide useful information about the involvement of genes not yet identified through biochemical approaches. Human MDMs were generated from peripheral blood mononuclear cells (PBMCs) and infected with a recombinant adenovirus expressing Vpr, and analyzed by cDNA microarray. HIV-1 Vpr protein induced interferon (IFN)-stimulated genes (ISGs) such as *IRF7*, and caused phosphorylation of STAT1 at tyrosine 701 in human MDMs. These findings enhance the current understanding of HIV-1 replication and pathogenesis in human macrophages.

## Results

### Expression of Vpr and ZsGreen1 in human MDMs

**Figure 1:**
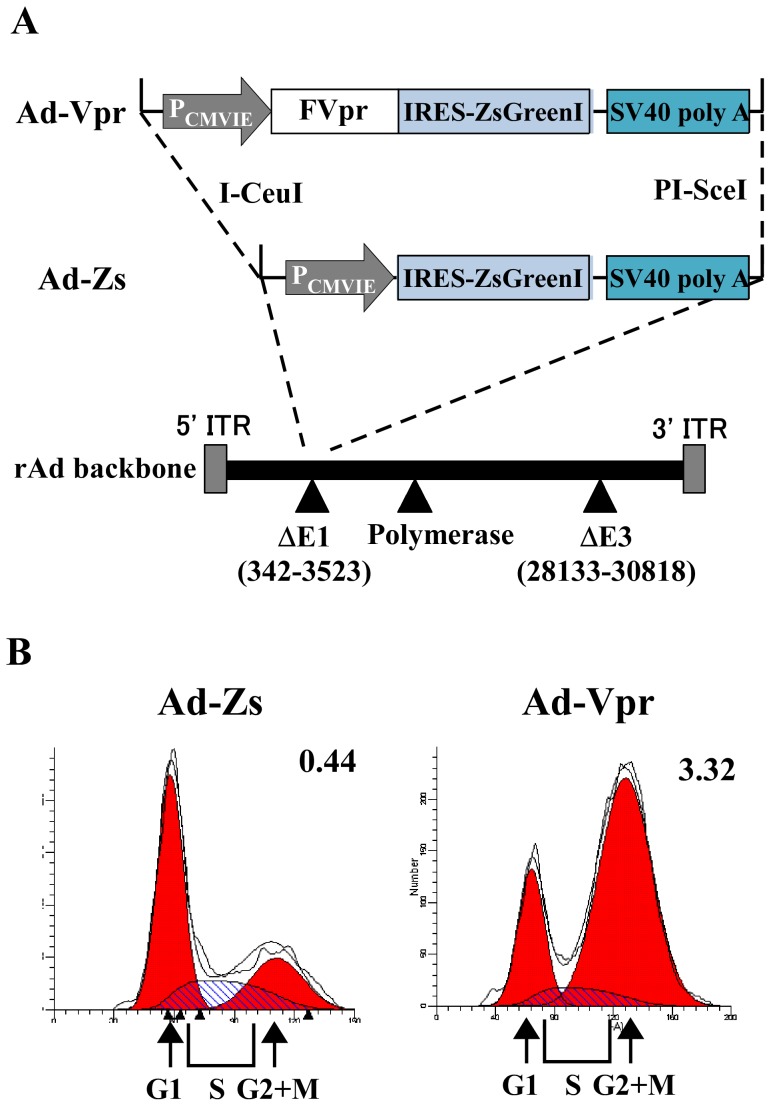
Schematic diagram of the Ad-Vpr and Ad-Zs vectors and analysis of their functional expression. (A) Recombinant adenovirus vectors expressing either FLAG-Vpr and ZsGreen1 or ZsGreen1 were generated using the Adeno-X™ expression system, as described in Materials and Methods. The transgene cassettes that replace the deleted E1 region contain a cytomegalovirus (CMV) promoter driving the expression of FLAG-Vpr and ZsGreen1 or ZsGreen1 protein, followed by an SV40 polyadenylation signal. The solid triangles indicate the regions deleted in the recombinant adenovirus (rAd) backbone. ITR: Inverted terminal repeats. (B) HeLa cells were infected with Ad-Vpr or Ad-Zs at MOI 50. At 48 h post-infection, cells were fixed and stained with propidium iodide for the analysis of DNA content. ZsGreen1-positive cells were analyzed by flow cytometry using Cell Quest for acquisition and ModFit LT. Arrowheads indicate peaks representing cells in the G1 and G2+M phases. The G2+M: G1 ratio is indicated in the upper right of each graph.

To better understand the role of HIV-1 Vpr protein in human MDMs, a recombinant adenovirus expressing ZsGreen1 and FLAG-tagged Vpr, Ad-Vpr, was generated. As a control, a recombinant adenovirus expressing ZsGreen1, Ad-Zs, was used. A schematic diagram of both recombinant adenoviruses is shown in Figure 1A. To examine whether Vpr induces cell-cycle arrest at the G2 phase, HeLa cells were infected with Ad-Vpr or Ad-Zs at a multiplicity of infection (MOI) of 50. At 48 h post-infection, cells were harvested for analysis of DNA content and stained with propidium iodide (PI). The DNA content of ZsGreen1-positive cells was analyzed by flow cytometry, which revealed a dramatic increase in the proportion of cells in the G2 phase of the cell cycle in cells infected with Ad-Vpr (21.22% and 70.37% were in the G1 and the G2+M phases, respectively, and the G2+M: G1 ratio was 3.32) compared to cells infected with the control Ad-Zs (54.06% and 23.87% were in the G1 and G2/M phases, respectively, and the G2+M: G1 ratio was 0.44) (Figure 1B). These results indicate that the recombinant adenovirus expressing FLAG-Vpr induces G2 cell-cycle arrest.

**Figure 2:**
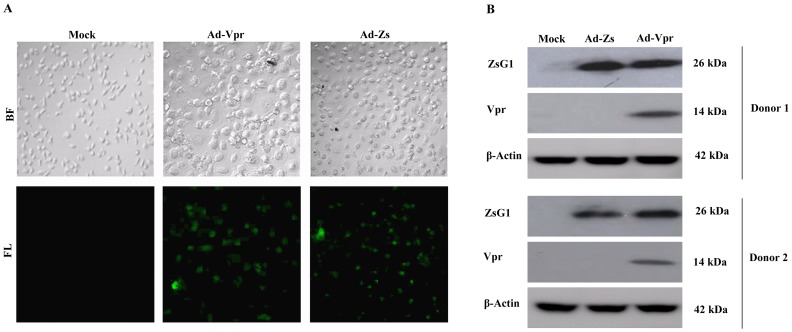
Expression analyses of HIV-1 Vpr protein in human monocyte-derived macrophages (MDMs). (A) Peripheral blood mononuclear cells (PBMCs) were isolated from two healthy donors through leukophoresis, cultured *in vitro*, and differentiated into MDMs as described in Materials and Methods. At day 7, the MDMs were infected with either Ad-Vpr or Ad-Zs, or were left untreated as mock-infected controls (left). At 48 h post-infection, the cells from Donor 1 were visualized by fluorescence (FL) and bright field phase contrast (BF) microscopy. (B) The cells from the two donors (upper panel, Donor 1; lower panel, Donor 2) were lysed and subjected to Western blot analyses using Vpr, ZsGreen1, and β-actin antibodies.

Purified and titrated Ad-Vpr and Ad-Zs were next used to infect MDMs derived from peripheral blood monocytes from two normal healthy donors (Figure 2). PBMCs were isolated from heparinized whole blood from two healthy donors by standard density gradient centrifugation with Ficoll-Paque. PBMCs were harvested from the interface and CD14^+^ cells were separated by high-gradient magnetic sorting using MACS beads. The isolated CD14^+^ cells were differentiated into MDMs for 7 days, and then infected with the Ad-Vpr or Ad-Zs at a MOI of 100. After 48 h, the cells were either observed under a fluorescence microscope or lysed and analyzed for the expression of Vpr and ZsGreen1 protein by Western blotting. Fluorescence microscopy showed that ZsGreen1 was expressed in both Ad-Vpr- and Ad-Zs-infected MDMs compared to mock-infected controls, which remained ZsGreen1-negative (Figure 2A). As shown in Figure 2B, a 26 kDa band representing ZsGreen1 and a 14 kDa band representing Vpr was detected; these apparent molecular masses are consistent with their respective predicted sequences. Further, there was no difference in ZsGreen1 expression between the two populations of MDMs (Figure 2B). These results confirm the suitability of the adenovirus-infected MDMs for downstream assays.

### Microarray analysis of MDMs infected with Ad-Vpr or Ad-Zs

**Figure 3:**
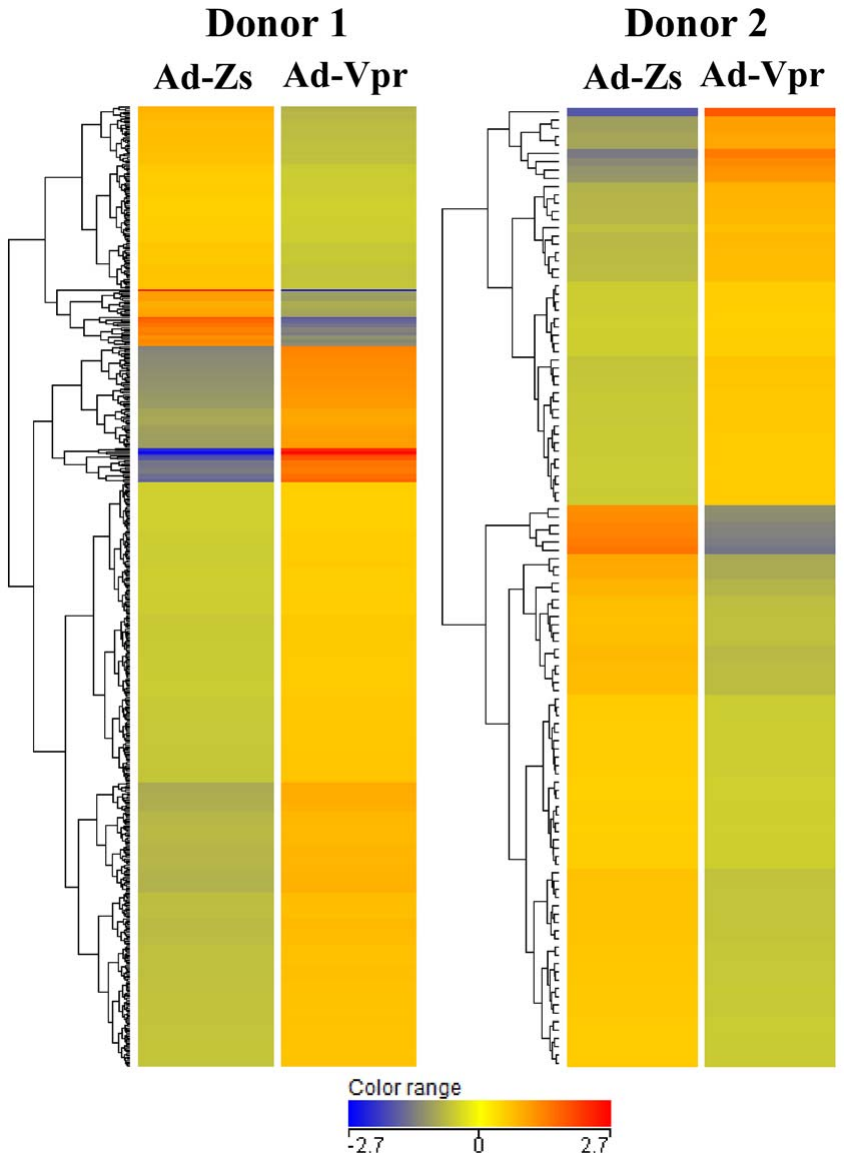
Differential expression profiling of cellular genes after infection with Ad-Vpr in human monocyte-derived macrophages (MDMs). Heat map of hierarchical gene clustering showing all genes that were either up- or down-regulated (>2-fold change) upon Ad-Vpr infection in MDMs from both donors. The color represents the normalized expression of genes in MDMs infected with Ad-Vpr or Ad-Zs (see color key). Gene up-regulation is denoted in red and gene down-regulation is denoted in blue.

To evaluate changes in the expression of host cellular genes in response to HIV-1 Vpr protein, Ad-Vpr- and Ad-Zs-infected macrophages were subjected to cDNA microarray analyses using a commercially available Affymetrix GeneChip oligonucleotide array (Human Genome U133 Plus 2.0), which interrogates more than 47,000 transcripts from 38,500 genes. This approach enabled us to monitor Vpr-induced changes in the global gene profile of the MDMs. Data analysis using GeneSpring GX software showed that Vpr modulated the expression of 557 genes in Donor 1 and 116 genes in Donor 2. Given that the array analyzes more than 47,000 gene transcripts, this is considered a minor change in the global host gene profiles (Figure 3). Heat maps from both donors (Figure 3) show that the global gene expression profiles were different in each donor, indicating that there is individual variability in the response to Vpr at the transcriptional level.

**Figure 4:**
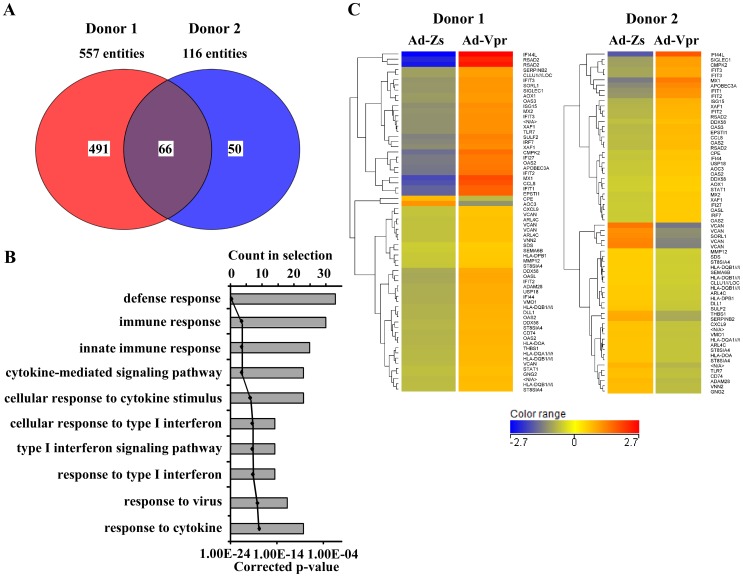
Gene ontology of differentially expressed genes after infection of human monocyte-derived macrophages (MDMs) with Ad-Vpr. (A) Venn diagram representing the number of differentially expressed cellular genes (>2-fold change in both donors) after infection of human MDMs with Ad-Vpr. (B) The top ten genes ontology classified by corrected p-value, and (C) heat map of hierarchical gene clustering of the 66 differentially regulated in both donors. Gene up-regulation is denoted in red and gene down-regulation is denoted in blue.

The differentially regulated genes were filtered to determine gene entities common to both donors. Out of 557 genes altered in response to Vpr in Donor 1 and 116 genes in Donor 2, only 66 genes were common to both (Figure 4A). Gene ontology was ranked based on the corrected p-values. The ten most significant pathways common to both donors are shown in Figure 4B. HIV-1 Vpr significantly altered the expression profiles of cellular genes mainly involved in the innate immune response, type I IFN signaling, and cytokine-mediated signaling. A complete list of all 66 genes common to both donors is shown in the form of heat maps in Figure 4C.

**Figure 5:**
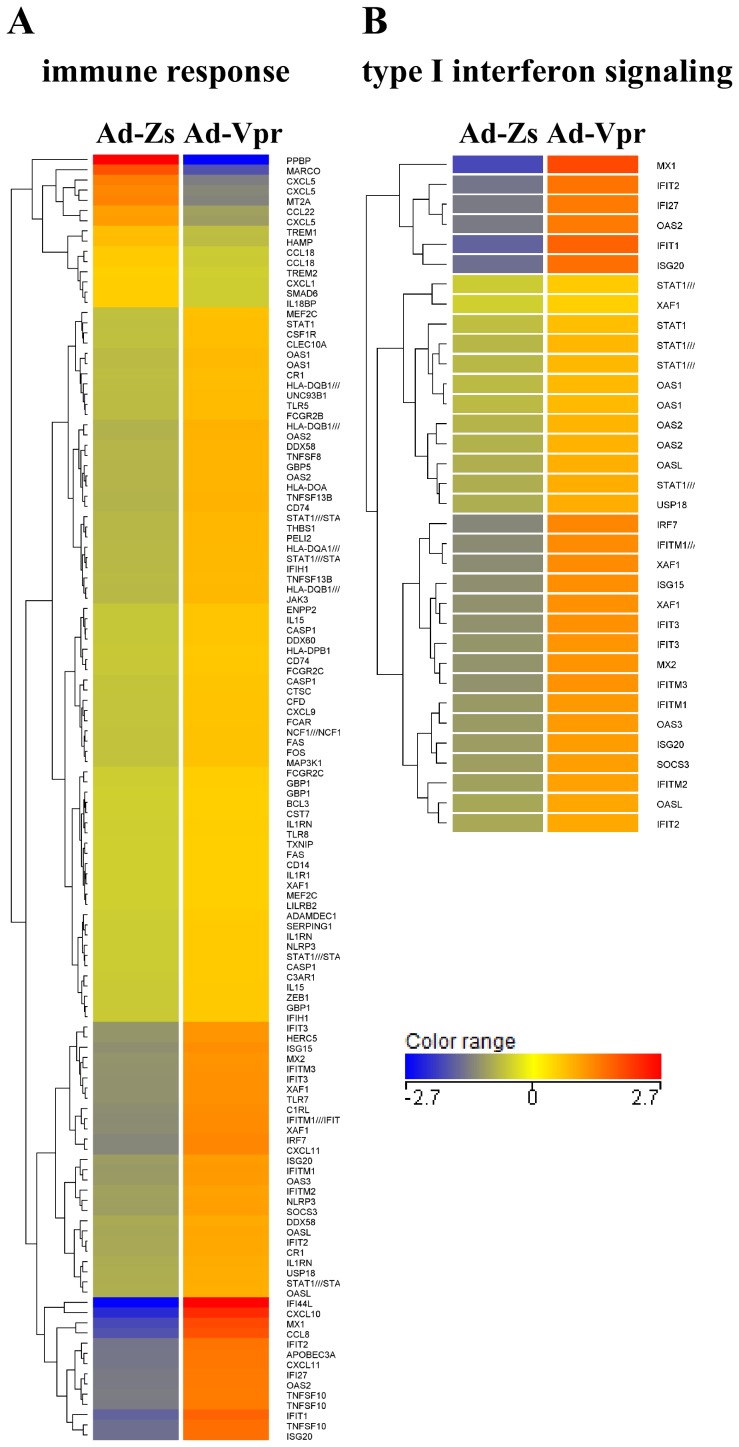
Differential expression profiling of cellular genes involved in the immune response and the type I interferon pathway after infection with Ad-Vpr in human monocyte-derived macrophages (MDMs) from Donor 1. Heat map showing genes related to the immune response (left: GO: 0006955) and the type I interferon signaling (right: GO: 0060337) that were either up- or down-regulated (>2-fold change) upon Ad-Vpr infection of MDMs from Donor 1. The color coding represents the normalized expression of genes in MDMs infected with Ad-Vpr or Ad-Zs (see color key). Gene up-regulation is denoted in red and gene down-regulation is denoted in blue.

Most of the altered genes were involved in the immune response or the defense response (Figure 4B); therefore, genes related to the immune response (GO: 0006955) were further analysed. A complete list of the 126 and 41 genes differentially regulated in Donor 1 and Donor 2 respectively, is shown in Table 1. A significant majority of the up-regulated genes are involved in the immune response. *IFI44L* (40-fold), *CXCL10* (23-fold), *MX1* (15-fold), *CCL8* (13-fold), *IFIT1* (10-fold), *TNFSF10* (TRAIL) (8-fold), *ISG20* (8-fold), *IFIT2* (8-fold), *APOBEC3A* (7-fold), *CXCL11* (7-fold), *IFI27* (7-fold), *OAS2* (7-fold), *IRF7* (6-fold), and *ISG15* (5-fold) were the most highly up-regulated genes, whereas *PPBP*(CXCL7) (96-fold), *MARCO* (13-fold), *CXCL5* (7-fold), *MT2A* (6-fold), and *CCL22* (4-fold) were the most highly down-regulated genes in Donor 1 (Table 1). In contrast, *IFI44L* (12-fold), *MX1* (7-fold), *APOBEC3A* (6-fold), *IFIT1* (5-fold), *IFIT2* (4.5-fold), *IFIT3* (4-fold), *ISG15* (3-fold), *XAF1* (3-fold), *OAS3* (3-fold), *CCL8* (3-fold), *OAS2* (3-fold), *DDX58* (2.5-fold), *STAT1* (2-fold), *MX2* (2-fold), *IRF7* (2-fold) and *CCL2* (2-fold) were the most highly up-regulated genes, whereas *THBS1* (3-fold), *HLA-DQA* (3-fold), *TLR7* (2.6-fold), *CD74* (2.5-fold), *CXCL2* (2-fold), *CCR2* (2-fold) and *CXCL9* (2-fold) were the most highly down-regulated genes in Donor 2 (Table 1). By close examination of the data set (Figure 5 and Table 1), it was observed that several ISGs, which are mainly produced in response to type I interferon [27], were up-regulated in the Vpr-expressing MDMs. A hierarchical heat map of all the genes up-regulated in Donor 1 (>2.0-fold change) that are related to the immune response and type 1 IFN signalling is shown in Figure 5A and B. Collectively, microarray analyses indicate that HIV-1 Vpr leads to the differential regulation of genes involved in innate immunity, type I IFNs, cytokine production, and cell signalling, resulting in activation of antiviral responses in MDMs.

**Table 1:** Differentially expressed genes (fold change >2.0) associated with immune response (GO: 0006955) upon Ad-Vpr infection in Donor 1 and Donor 2.

Probe Set ID	Gene Symbol	Entrez Gene	Fold Change		Regulation
			Donor 1	Donor 2	
214146_s_at	PPBP	5473	−96.30		Down
205819_at	MARCO	8685	−12.69		Down
215101_s_at	CXCL5	6374	−6.77		Down
207852_at	CXCL5	6374	−5.81		Down
212185_x_at	MT2A	4502	−6.16		Down
207861_at	CCL22	6367	−4.06		Down
214974_x_at	CXCL5	6374	−4.23		Down
219434_at	TREM1	54210	−2.61		Down
220491_at	HAMP	57817	−2.66		Down
209924_at	CCL18	6362	−2.15		Down
32128_at	CCL18	6362	−2.17		Down
219725_at	TREM2	54209	−2.01		Down
204470_at	CXCL1	2919	−2.10		Down
207069_s_at	SMAD6	4091	−2.07		Down
222868_s_at	IL18BP	10068	−2.07		Down
209200_at	MEF2C	4208	2.52		Up
209969_s_at	STAT1	6772	2.59	2.03	Up
203104_at	CSF1R	1436	2.54		Up
206682_at	CLEC10A	10462	2.56		Up
202869_at	OAS1	4938	2.75		Up
205552_s_at	OAS1	4938	2.75		Up
217552_x_at	CR1	1378	2.63		Up
211656_x_at	HLA-DQB1	3119	2.68	−2.06	Up/down
225869_s_at	UNC93B1	81622	2.70		Up
210166_at	TLR5	7100	2.70		Up
210889_s_at	FCGR2B	2213	2.70		Up
212998_x_at	HLA-DQB1	3119	3.12	−2.14	Up/down
228607_at	OAS2	4939	3.10	2.15	Up
222793_at	DDX58	23586	2.96	2.09	Up
235735_at	TNFSF8	944	2.97		Up
238581_at	GBP5	115362	3.00		Up
206553_at	OAS2	4939	3.01	2.24	Up
226878_at	HLA-DOA	3111	3.01	−2.37	Up
223502_s_at	TNFSF13B	10673	3.03		Up
1567628_at	CD74	972	3.04	−2.54	Up/down
M97935_MA_at	STAT1	6772	2.88		Up
201110_s_at	THBS1	7057	2.86	−3.44	Up/down
219132_at	PELI2	57161	2.86		Up
212671_s_at	HLA-DQA1	3117	2.84	−2.40	Up
M97935_MB_at	STAT1	6772	2.84		Up
1555464_at	IFIH1	64135	2.84		Up
223501_at	TNFSF13B	10673	2.80		Up
209823_x_at	HLA-DQB1	3119	2.82	−2.07	Up/down
227677_at	JAK3	3718	2.82		Up
209392_at	ENPP2	5168	2.32		Up
205992_s_at	IL15	3600	2.31		Up
211367_s_at	CASP1	834	2.31		Up
218986_s_at	DDX60	55601	2.31		Up
244485_at	HLA-DPB1	3115	2.25	−2.19	Up
209619_at	CD74	972	2.28		Up
211395_x_at	FCGR2C	9103	2.28		Up
206011_at	CASP1	834	2.36		Up
231234_at	CTSC	1075	2.38		Up
205382_s_at	CFD	1675	2.40		Up
203915_at	CXCL9	4283	2.43	−2.44	Up/down
207674_at	FCAR	2204	2.42		Up
204961_s_at	NCF1	653361	2.44		Up
215719_x_at	FAS	355	2.44		Up
209189_at	FOS	2353	2.45		Up
214786_at	MAP3K1	4214	2.45		Up
210992_x_at	FCGR2C	9103	2.07		Up
231577_s_at	GBP1	2633	2.09		Up
202269_x_at	GBP1	2633	2.03		Up
204908_s_at	BCL3	602	2.04		Up
210140_at	CST7	8530	2.04		Up
216243_s_at	IL1RN	3557	2.04		Up
220832_at	TLR8	51311	2.05		Up
201008_s_at	TXNIP	10628	2.03		Up
216252_x_at	FAS	355	2.02		Up
201743_at	CD14	929	2.02		Up
202948_at	IL1R1	3554	2.01		Up
242234_at	XAF1	54739	2.01		Up
209199_s_at	MEF2C	4208	2.01		Up
210146_x_at	LILRB2	10288	2.01		Up
206134_at	ADAMDEC1	27299	2.12		Up
200986_at	SERPING1	710	2.13		Up
212659_s_at	IL1RN	3557	2.13		Up
216015_s_at	NLRP3	114548	2.15		Up
M97935_3_at	STAT1	6772	2.14		Up
211368_s_at	CASP1	834	2.15		Up
209906_at	C3AR1	719	2.18		Up
217371_s_at	IL15	3600	2.20		Up
212764_at	ZEB1	6935	2.23		Up
202270_at	GBP1	2633	2.22		Up
219209_at	IFIH1	64135	2.22		Up
204747_at	IFIT3	3437	4.73	3.70	Up
219863_at	HERC5	51191	4.71		Up
205483_s_at	ISG15	9636	5.16	3.16	Up
204994_at	MX2	4600	4.88	2.20	Up
212203_x_at	IFITM3	10410	4.93		Up
229450_at	IFIT3	3437	5.05	3.70	Up
206133_at	XAF1	54739	4.99	2.19	Up
220146_at	TLR7	51284	5.00	−2.58	Up/down
218983_at	C1RL	51279	5.32		Up
201601_x_at	IFITM1	10581	5.46		Up
228617_at	XAF1	54739	5.44	3.02	Up
208436_s_at	IRF7	3665	5.87	2.16	Up
210163_at	CXCL11	6373	5.89		Up
33304_at	ISG20	3669	4.29		Up
214022_s_at	IFITM1	8519	4.46		Up
218400_at	OAS3	4940	4.41	2.82	Up
201315_x_at	IFITM2	10581	3.97		Up
207075_at	NLRP3	114548	4.09		Up
227697_at	SOCS3	9021	4.17		Up
218943_s_at	DDX58	23586	3.49	2.54	Up
205660_at	OASL	8638	3.65	2.13	Up
217502_at	IFIT2	3433	3.59	2.93	Up
244313_at	CR1	1378	3.59		Up
216244_at	IL1RN	3557	3.36		Up
219211_at	USP18	11274	3.32	2.26	Up
M97935_5_at	STAT1	6772	3.28		Up
210797_s_at	OASL	8638	3.23		Up
204439_at	IFI44L	10964	40.44	11.7	Up
204533_at	CXCL10	3627	22.91		Up
202086_at	MX1	4599	14.65	7.07	Up
214038_at	CCL8	6355	12.93	2.68	Up
226757_at	IFIT2	3433	7.64	4.52	Up
210873_x_at	APOBEC3A	200315	7.49	5.67	Up
211122_s_at	CXCL11	6373	7.45		Up
202411_at	IFI27	3429	7.03	2.14	Up
204972_at	OAS2	4939	7.05	2.72	Up
202687_s_at	TNFSF10	8743	6.85		Up
214329_x_at	TNFSF10	8743	6.95		Up
203153_at	IFIT1	3434	10.06	4.89	Up
202688_at	TNFSF10	8743	8.34		Up
204698_at	ISG20	3669	8.42		Up
216598_s_at	CCL2	6347		2.16	Up
236203_at	HLA-DQA	100507718		−3.46	Down
213831_at	HLA-DQA	100507718		−2.12	Down
209480_at	HLADQB	3119		−2.03	Down
209774_at	CXCL2	2920		−2.06	Down
211743_s_at	PRG2	5553		−2.40	Down
206978_at	CCR2	729230		−2.19	Down

### Validation of the expression of host genes involved in the type 1 IFN pathway by real-time quantitative reverse transcription-polymerase chain reaction (qRT-PCR)

**Figure 6:**
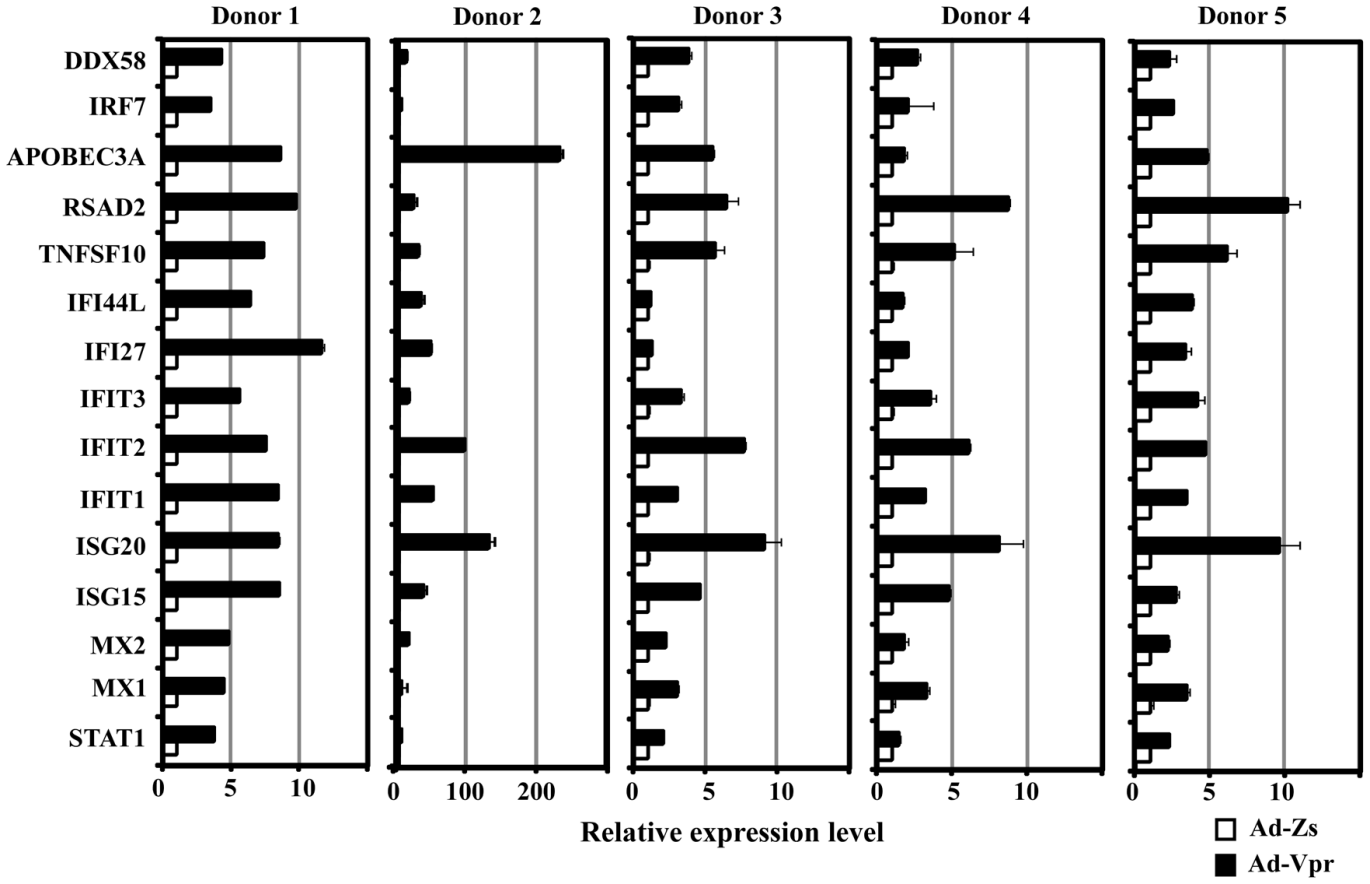
Validation of microarray data by qRT-PCR. Peripheral blood mononuclear cells (PBMCs) isolated from Donor 1, Donor 2 and three other healthy donors (Donors 3–5) through leukophoresis were cultured *in vitro* and differentiated into human MDMs as described in Materials and Methods. At day 7, the MDMs were infected with Ad-Vpr or Ad-Zs. At 48 h post-infection, RNA was extracted and subjected to qRT-PCR to amplify the selected genes using specific primers. Relative mRNA levels of the indicated genes are shown. Values are expressed as the fold change in Ad-Vpr-infected cells compared to Ad-Zs-infected cells and normalized to the expression of a housekeeping gene (*GAPDH*). The results represent the mean ± standard deviation (SD) of three samples from one experiment (P<0.05).

Validation of the results obtained by microarray analysis was performed by qRT-PCR evaluating the mRNA levels of selected up-regulated genes involved in the immune response. Genes were selected for confirmation either because they were known to be induced in response to type I IFN and reportedly involved in the innate immune antiviral response [27], [28] or because they were common to both donors. The transcriptional levels of 15 genes were measured in Donor 1 by qRT-PCR with the primers listed in Table 2, using *GAPDH* as an internal control. In general, there was a strong correlation between the microarray data and the qRT-PCR data at 48 h post-infection; the two techniques yielded very similar expression profiles for all 15 genes in Donor 1 (Figure 6 and Table 1). However, there were some discrepancies, e.g., the qRT-PCR results showed a slightly higher increase than the microarray analysis for *IFI27*. Similarly, the expression levels of *IFIT1*, *IFI44L*, *MX1*, and *RSAD2* (which encodes the viperin protein) were higher in the microarray data compared to their respective relative expression levels in the qRT-PCR data. On the other hand, in Donor 2 except for *IRF7* (4-fold) and *MX1* (5-fold) higher expression levels of *APOBEC3A* (233-fold), *ISG20* (132-fold), *IFIT2* (97-fold), *IFIT1* (51-fold), *ISG15* (38-fold), *IFI27* (47-fold), *IFI44L* (36-fold), *TNFSF10* (*TRAIL*) (29-fold), *RSAD2* (22-fold), *MX2* (17-fold), *IFIT3* (16-fold), *DDX58* (12-fold) and *STAT1* (5-fold) were observed by qRT-PCR compared to their respective microarray data (Figure 6 and Table 1). These inconsistencies were probably due to the differences in transcripts variants or due to the intrinsic differences between the two techniques, notably in the normalization methods. For microarray experiments, the normalization was based on a large number of genes, whereas in the qRT-PCR experiments, a single housekeeping gene was used as an internal control against which the results were normalized. Overall, the qRT-PCR results were in agreement with the array data i.e. differential up-regulation giving us strong confidence in the interpretation of the gene expression data obtained through microarray.

**Table 2:** Primers used for real-time PCR.

Name	5′ Sequence	3′ Sequence
STAT1	CCATCCTTTGGTACAACATGC	TGCACATGGTGGAGTCAGG
MX1	CAGCACCTGATGGCCTATCA	ACGTCTGGAGCATGAAGAACTG
MX2	AAACTGTTCAGAGCACGATTGAAG	ACCATCTGCTCCATTCTGAACTG
ISG15	ACTCATCTTTGCCAGTACAGGAG	CAGCATCTTCACCGTCAGGTC
ISG20	TCACCCCTCAGCACATGGT	TTCAGGAGCTGCAGGATCTCTAG
IFIT1	GCAGCCAAGTTTTACCGAAG	GCCCTATCTGGTGATGCAGT
IFIT2	CGAACAGCTGAGAATTGCAC	CAAGTTCCAGGTGAAATGGC
IFIT3	AGTCTAGTCACTTGGGGAAAC	ATAAATCTGAGCATCTGAGAGTC
IFI27	GGCAGCCTTGTGGCTACTCT	ATGGAGCCCAGGATGAACTTG
IFI44L	GTATAGCATATGTGGCCTTGCTTACT	ATGACCCGGCTTTGAGAAGTC
TNFSF10	GAGCTGAAGCAGATGCAGGAC	TGACGGAGTTGCCACTTGACT
RSAD2	AGGTTCTGCAAAGTAGAGTTGC	GATCAGGCTTCCATTGCTC
APOBEC3A	GAGAAGGGACAAGCACATGG	GTCTTATGCCTTCCAATGCC
IRF7	TACCATCTACCTGGGCTTCG	AGGGTTCCAGCTTCACCA
DDX58	ATCCCAGTGTATGAACAGCAG	GCCTGTAACTCTATACCCATGTC
GAPDH	ACAGTCAGCCGCATCTTCTTTTGC	TTGAGGTCAATGAAGGGGTC

Next, to demonstrate whether similar results could be obtained in other healthy donors, the transcriptional levels of these 15 genes were measured in MDMs derived from three additional healthy donors (Donors 3–5) by qRT-PCR. As shown in Figure 6, the expression profiles of the three additional donors were generally consistent with the data obtained from Donor 1. However, the expression levels of the *IFI27* and *IFI44L* genes, which were up-regulated approximately 7-fold and 40-fold, respectively, in the presence of Vpr in MDMs derived from Donor 1, were only slightly up-regulated in Donors 3 and 4. These results indicated that the activation of the type I IFN pathway was common to all the tested healthy donors.

### Confirmation of protein expression by Western blotting

**Figure 7:**
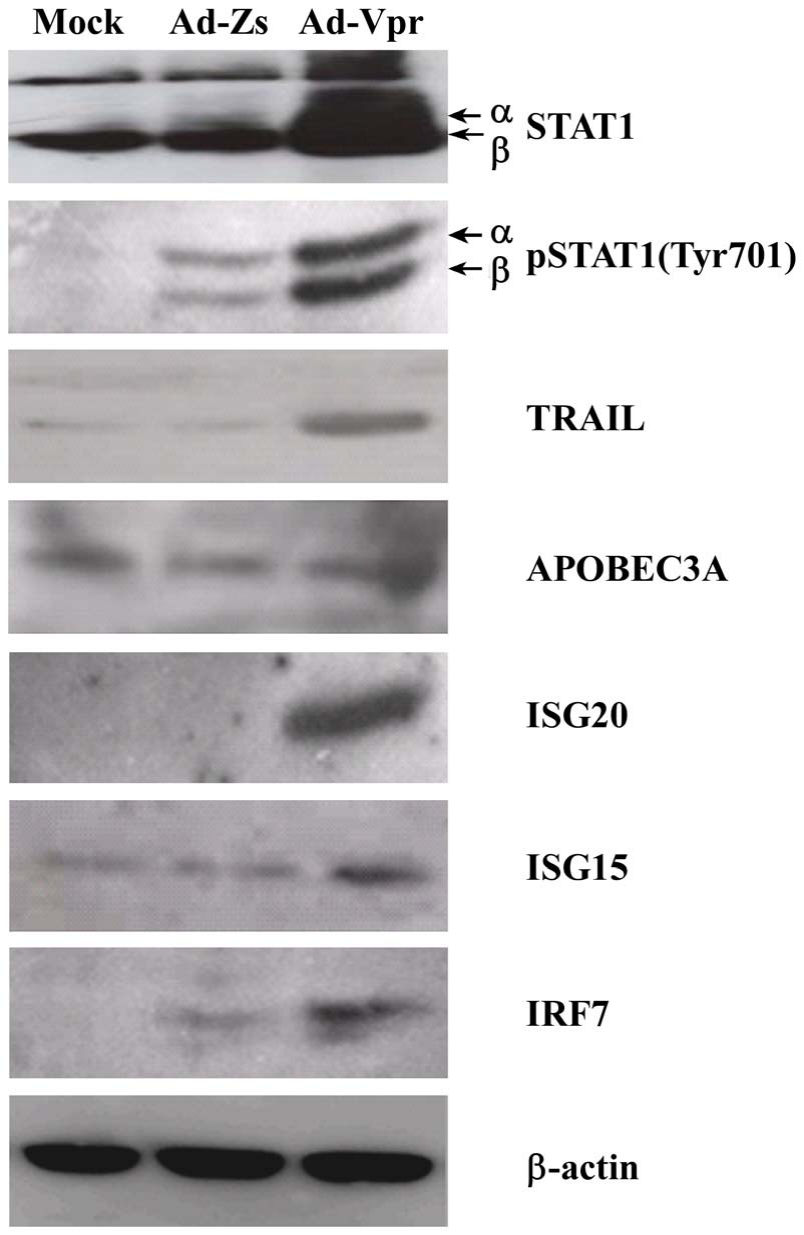
Validation of differentially expressed genes at the protein level. Human monocyte-derived macrophages (MDMs) were infected with Ad-Vpr or Ad-Zs, or mock-infected as a control. At 48 h post-infection, the cells were washed, lysed, and subjected to Western blot analyses with the indicated antibodies. A β-actin antibody was used as a loading control.

Finally, Western blotting was performed to examine the effect of HIV-1 Vpr on the protein expression levels of IRF7, STAT1, ISG15, ISG20, APOBEC3A, and TRAIL in MDMs. Cell lysates were prepared from Ad-Vpr, Ad-Zs, or mock-infected MDMs and subjected to Western blotting using specific antibodies. β-actin was used as a loading control. Consistent with the microarray data and the qRT-PCR results, STAT1, ISG15, ISG20, IRF7, and TRAIL were up-regulated in Ad-Vpr-infected macrophages compared to Ad-Zs- or mock-infected controls (Figure 7); however, APOBEC3A, which was originally shown to be up-regulated at the transcriptional level by both microarray and real-time PCR, was not induced at the protein level compared to controls, as measured by Western blotting (Figure 7). Why the *APOBEC3A* gene transcript failed to express its gene product is not clear; however, differential regulation of gene transcription does not ensure a corresponding change in gene product levels. Taken together, these results clearly indicate that HIV-1 Vpr protein leads to the activation of the type I IFN pathway and the subsequent up-regulation of various ISGs in human MDMs.

## Discussion

The data presented herein are the first analysis of the changes in gene transcription that occur following *in vitro* infection of human MDMs with an adenovirus expressing HIV-1 Vpr protein. Although some previous studies have shown that HIV-1 infection leads to the activation of innate immunity and thus the induction of various ISGs in human MDMs [5], [29]–[37], the specific role of Vpr in the induction of ISGs in human MDMs has not been documented.

In this study, by utilizing an Affymetrix oligonucleotide microarray, we demonstrated that the majority of the genes differentially regulated by Ad-Vpr in both donors were involved in the immune response, indicating the important role played by HIV-1 Vpr protein in human MDMs. A large number of genes from this group is predicted to be activated during the innate immune response (Table 1) as part of the host defense response to clear viral infections [27], [28], [38]. We observed an increase in the levels of various ISGs such as *MX1, IFI44L, DDX58, RSAD2*, and several of the *IFITs*, which have been shown to play an important role against HIV-1 infection in MDMs [31]. MX2 has recently been reported to be an IFN-induced inhibitor of HIV-1 infection in human monocytoid cell lines [39]. Since the differential expression levels of the *MX1, MX2, IFIT1, IFIT2, IFIT3, IFIT27, IFI44L, DDX58*, and *RSAD2* genes obtained through microarray strongly correlated with the real-time PCR data, it is reasonable to speculate that the expression of these proteins may be up-regulated following HIV-1 infection in human MDMs.

Real-time PCR data and Western blot analysis confirmed the activation of IRF7 by HIV-1 Vpr in human MDMs (Figures 6 and 7). IRF7 is the master regulator of type I IFN-dependent immune responses [40] and plays an important role in HIV-1 pathogenesis [34]. IRF7 promotes autocrine and paracrine activation of STAT1 and plays a critical role in virus-mediated induction of IFN-α [41]. It is known that type I IFNs activate the Janus kinases (JAKs) and the STAT transcription factors, which ultimately leads to the expression of target genes [42], [43]. The *STAT1* gene encodes a 91-kDa protein which is activated by both type I and type II IFNs [44]. This important transcription factor is phosphorylated by the JAKs in response to proinflammatory and regulatory factors [38]. It has been shown that the STAT1 pathway plays an important role in the pathogenesis of HIV-1 infection [45], [46]; indeed, activation of the STAT1 pathway by HIV-1 Vpr is demonstrated in this study. We further showed that in the presence of Vpr protein the level of STAT1 phosphorylation at tyrosine 701 is much higher than the control recombinant adenovirus (Figure 7). The exact mechanism through which Vpr leads to the phosphorylation of STAT1 at tyrosine 701 is not known and requires further study.

HIV-1 Vpr protein caused the up-regulation of various ISGs, such as ISG15 and ISG20 (Figures 6 and 7), which can inhibit virus replication through different mechanisms [27], [28]. Previously, it has been shown that HIV-1 Vpr protein activates NF-κB [47], which might explain the up-regulation of various ISGs in our study. The ISGs act through a variety of mechanisms to render cells resistant to viral infection [27]. It has been shown that ISG15 is induced in HIV-1-infected MDMs [27], where it restricts and impedes HIV-1 replication by causing ISGylation of viral Gag protein and certain cellular factors [33]. Similarly, ISG20 has been shown to exhibit antiviral activity against HIV-1 [48]. Induction and activation of ISGs such as ISG15, ISG20, the IFITs, and viperin are thought to be the reason MDMs are relatively resistant to cell death and can act as long-term carriers of HIV-1 [31]. The observation that these genes were up-regulated in Ad-Vpr-infected MDMs in our study suggests that in HIV-1-infected macrophages, Vpr is responsible for the induction of these ISGs; thus due to these ISGs, macrophages are relatively resistant to Vpr-induced cell death.

TRAIL protein is produced after HIV-1 infection in monocytes due to the IFNα/β-mediated activation of the STAT1 signaling cascade [49], and has been shown to cause apoptosis in several cell lines during HIV-1 infection. Although an initial increase in TRAIL protein was shown to kill HIV-1-infected macrophages [50], the exact role of TRAIL-mediated apoptosis in the elimination of HIV-1-infected cells is not known. Here, we have shown that HIV-1 Vpr protein caused elevated levels of TRAIL protein in macrophages (Figures 6 and 7), which would presumably help to eliminate HIV-1-infected cells through TRAIL-mediated cell death [29], [50]–[52].

Our findings further demonstrated that HV-1 Vpr differentially regulated the expression levels of chemotactic cytokines such as *CXCL1, CXCL5, CXCL7, CXCL9, CXCL10*, and *CXCL11* (Table 1). A previous report has shown that *CXCL10* and *CXCL11* are up-regulated in HIV-1-infected macrophages and play a key role in the recruitment and spread of HIV-1 to susceptible CD4^+^ T-cells [53]. Surprisingly, our microarray data also showed that *CXCL10* and *CXCL11* were up-regulated in MDMs, by 23-fold and 7-fold, respectively (Table 1). Whether HIV-1 Vpr has a role in HIV-1 dissemination and the mechanism through which Vpr leads to the differential regulation of these chemokines in MDMs is not known. However, the recruitment of susceptible T-cells by HIV-1-infected human macrophages and the role of CXCL10 and CXCL11 will be intriguing to investigate in future studies.

HIV-1 Vpr is essential for efficient infection of non-dividing cells such as macrophages. It has been shown that HIV-1 Vpr is expressed within infected cells and is packaged into HIV-1 virions. Although, the virion-associated Vpr is able to cause cell cycle arrest of CD4^+^ T cells in vivo [54], the induction of ISGs by this biologically active form of Vpr is not known. However, our recent studies have confirmed that the induction of ISGs in HIV-1_AD8_/Vpr^+^ infected MDMs (Unpublished results) is similar to ISGs induced by Vpr in Ad-Vpr infected MDMs. Our data indicating that Vpr leads to the induction of ISGs and activation of innate immune responses is contrary to some of the previously published reports which showed that Vpr helps HIV-1 to escape the innate immune responses by either counteracting the UNG2, a host cellular intrinsic factor which inhibits HIV-1 replication [55]–[57] or by manipulating the cellular SLX4 complex which is a negative regulator of Type 1 IFN production [58]. Therefore, the complex role played by Vpr in escaping HIV-1 virions from innate immune responses or by activating innate immunity through inducing ISGs in HIV-1 infected macrophages must be investigated in future studies.

Our data confirmed that HIV-1 Vpr leads to the induction of ISGs in MDMs. However, our findings showed some donor-specific differences in the expression profiles of these ISGs, which might be due to differences in overall susceptibility and the host response to the HIV-1 Vpr infection. These differences and the number of donors used in the study should not be considered a limiting factor because the expression profiles of the selected genes in all donors were independently confirmed by qRT-PCR with reproducible and consistent readouts each time (Figure 6). Furthermore, we cannot rule out the possibility that some of these ISGs are regulated by direct or indirect interactions of Vpr with cellular proteins related to the innate immune response, including cellular transcription factors such as NF-κB, AP-1, and Sp-1 [1], [47].

In conclusion, our studies have identified IRF7, STAT1, ISG15, ISG20, and TRAIL as key up-regulated molecules in MDMs harboring HIV-1 Vpr. Based on previous published reports and our present data; we suggest a potential role for these genes in host defense against HIV-1 replication and infection. Future studies to elucidate the mechanisms through which Vpr up-regulates these molecules as well as their roles in HIV-1 pathogenesis will certainly improve our understanding of the replication and pathogenesis of the HIV-1.

## Materials and Methods

### Cell culture and preparation of human MDMs

Human cervical HeLa cells and human embryonic kidney HEK-293 cells were maintained in Dulbecco's modified Eagle's medium (DMEM; Life Technologies) supplemented with 10% heat-inactivated fetal bovine serum (FBS; Sigma) and 100 units/mL penicillin/streptomycin (Sigma). Plasmid transfection was performed using Lipofectamine 2000 (Life Technologies).

Human PBMCs were obtained using a standard Ficoll-Paque (Pharmacia) gradient from heparinized blood from healthy individuals. CD14^+^ cells were isolated by positive selection with anti-human CD14^+^ magnetic beads (MACS system; Miltenyi Biotec). Purity was greater than 95% (data not shown). Primary MDMs were generated by culturing CD14^+^ cells in RPMI 1640 medium (Sigma) supplemented with 10% FBS (Cell Culture Bioscience), 5% human AB serum(Sigma), antibiotics, and GlutaMax (Gibco), and containing recombinant human macrophage colony-stimulating factor (M-CSF; PeproTech). After 7 days, cellular differentiation status was confirmed by detection of MDM surface such as CD14 and CD68 (data not shown). All participants provided written informed consent. Ethics approval for this study was granted by the RIKEN Ethics Committees [Certificate No. Wako 21–2 (3)].

### Antibodies

STAT1 (#9172), phospho-STAT1 (Tyr701; #9171), and IRF-7 (#4920) rabbit polyclonal antibodies were from Cell Signaling Technology. The ISG15 mouse monoclonal antibody (MAb) (#AIS0701) was from ATGen. The TRAIL rabbit polyclonal antibody (#54008) was from ANASPEC. The ISG20 rabbit polyclonal antibody (#ARP40392-T100) was from Aviva System Biology. The HIV-1 Vpr mouse MAb #3 was produced by immunization of synthetic peptides N'-CQAPEDQGPQREPYN-C' corresponding to amino acids 3–16 of Vpr. The APOBEC3A goat polyclonal antibody (#NB100-93428) was from Novus Biologicals. ZsGreen1 rabbit polyclonal antibody (#632474) was from Clontech Laboratories. Fluorescein isothiocyanate (FITC)-conjugated MAbs directed against the human surface markers CD14 and CD68 were from Miltenyi Biotec and used at the supplier's recommended concentrations. The β-actin (#1978) MAb and *horseradish peroxidase* (HRP)-labeled donkey anti-goat or goat anti-mouse secondary antibodies were from Sigma.

### Generation of recombinant adenoviruses

Adenoviruses were constructed using the Adeno-X™ expression system (Clontech Laboratories). Briefly, wild-type (wt) Vpr from HIV-1_NL43_ [59] (GenBank accession no. M 19921) was PCR-amplified with the FLAG tag incorporated using the primers GAAGCTAGCGACTACAAGGATGACGATGACAAAATGGAACAAGCCCCAGAAGA (forward) and GCTCTAGACTAGGATCTACTGGCTCCAT (reverse), and cloned into the pShuttle2 vector at the *Nhe*I and *Xba*I restriction sites. Similarly, the ZsGreen1 gene was PCR-amplified with the FLAG tag incorporated using the primers TAATCTAGAGACTACAAGGATGACGATGACAAAGCCCCTCTCCCTCCCCCCCCCCTAA (forward) and TAGCGGCCGCTCAGGGCAAGGCGGAGCCGGAG (reverse) using the pRetroX-IRES2-ZsGreen1 plasmid (Clontech Laboratories) as a template, and then cloned into the pShuttle vector just downstream of Vpr at the *XbaI* and *Not*I restriction sites. The integrity of the generated recombinant plasmids was confirmed by DNA sequencing. Then the entire cassette (flanked by unique I-*Ceu*I and PI-*Sce*I restriction sites) was excised and ligated into Adeno-X viral DNA using the Adeno-X expression system 1, according to the manufacturer' s instructions (Clontech Laboratories).

Adeno-X viral DNA containing the FLAG-Vpr or ZsGreen1 was linearized with *Pac*I and transfected into HEK293 cells with Lipofectamine 2000 (Life Technologies). The recombinant adenoviruses were purified using the Adeno-X maxi purification kit (Clontech Laboratories) and titrated using the Adeno-X rapid titer kit (Clontech Laboratories), following the recommendations of the manufacturer. The virus stocks were stored at −80°C for future use.

### RNA extraction

MDMs were transduced with Ad-Vpr or Ad-Zs at a MOI of 100. The cells were harvested for RNA extraction at 48 h post-transduction. MDMs were washed three times with ice-cold PBS, and total RNA was extracted using the RNeasy mini kit with DNase digestion, according to the manufacturer's instructions (QIAGEN). RNA was quantified using a NanoDrop spectrophotometer (Thermo Fisher) and stored at −80°C. For microarray analysis, the quality of the RNA was determined using the Agilent Bioanalyzer (Agilent Technologies).

### Microarray and data analysis

RNA samples were analyzed by microarray using the GeneChip Human Genome U133 2.0 plus array (Affymetrix). Microarray hybridization and fluorescence detection were performed as described in the Affymetrix GeneChip Expression Analysis Technical Manual. The. cel data files generated by the Affymetrix microarray hybridization platform were analyzed using GeneSpring GX ver. 12.0 software (Agilent Technologies). Probe-level analysis was performed using the RMA algorithm. Microarray data have been deposited in NCBI's Gene Expression Omnibus and assigned the GEO Series accession number GSE56591. Fold changes in gene expression, hierarchical clustering, and gene ontology annotations were determined.

### Real-time qRT-PCR analysis of differentially expressed genes

Total RNA was prepared using the RNeasy mini kit as described above. RT-PCR was performed using specific primers and One-Step SYBR Green PCR mix (Takara), according to the manufacturer's manual. qRT-PCR was performed using a Prism 7500 sequence detection system (Applied Biosystems). Samples were run in triplicate and all data were normalized to *GAPDH* mRNA expression as an internal control.

### Western blotting

Mock or virus-infected MDMs were washed with PBS and then lysed with CelLytic™ MT Cell Lysis reagent (Sigma) which was supplemented with a protease inhibitor cocktail (Roche Diagnostics) according to the manufacturer's instructions. Protein concentrations were determined with a BCA protein assay kit (Pierce) using bovine serum albumin as a standard. Proteins were separated by sodium dodecyl sulfate-10% polyacrylamide gel electrophoresis (SDS-PAGE) and transferred to polyvinyl difluoride (PVDF; Millipore Corp.) membranes. The PVDF membranes were probed with the primary antibodies mentioned above followed by an anti-mouse HRP or anti-goat HRP or anti-rabbit HRP secondary antibody (Sigma), and signals were detected by enhanced chemiluminescence (GE Healthcare).

### Analysis of the cell cycle

HeLa cells were infected with an adenoviral vector expressing Vpr or expressing only ZsGreen1, as a control. At 48 h post-infection, the cells were harvested and fixed with 1% formaldehyde followed by 70% ethanol. Fixed cells were incubated in PBS containing RNase A (50 *µ*g/ml) at 37°C for 20 min and then stained with PI (40 *µ*g/ml). For each sample, at least 7,000 cells were analyzed using a FACS Calibur instrument (Becton-Dickinson) with CELL Quest software (Becton-Dickinson). Ratios of the numbers of cells in the G1 and G2/M phases (G2+M: G1 ratios) were calculated using ModFit LT Software (Verity Software House).

## References

[1] Herbein G, Gras G, Khan KA, Abbas W (2010) Macrophage signaling in HIV-1 infection. Retrovirology 7: 34. 20380698 10.1186/1742-4690-7-34 PMC2865443

[2] Kilareski EM, Shah S, Nonnemacher MR, Wigdahl B (2009) Regulation of HIV-1 transcription in cells of the monocyte-macrophage lineage. Retrovirology 6: 118. 20030845 10.1186/1742-4690-6-118 PMC2805609

[3] Barrero CA, Datta PK, Sen S, Deshmane S, Amini S, et al (2013) HIV-1 Vpr modulates macrophage metabolic pathways: a SILAC-based quantitative analysis. PLoS One 8: e68376. 23874603 10.1371/journal.pone.0068376 PMC3709966

[4] Carlson KA, Ciborowski P, Schellpeper CN, Biskup TM, Shen RF, et al (2004) Proteomic fingerprinting of HIV-1-infected human monocyte-derived macrophages: a preliminary report. J Neuroimmunol 147: 35–42. 14741425 10.1016/j.jneuroim.2003.10.039

[5] Van den Bergh R, Florence E, Vlieghe E, Boonefaes T, Grooten J, et al (2010) Transcriptome analysis of monocyte-HIV interactions. Retrovirology 7: 53. 20546557 10.1186/1742-4690-7-53 PMC2900222

[6] Tristem M, Marshall C, Karpas A, Hill F (1992) Evolution of the primate lentiviruses: evidence from vpx and vpr. Embo J 11, 3405–3412. 10.1002/j.1460-2075.1992.tb05419.x PMC556875 1324171

[7] Murakami T, Aida Y (2014) Visualizing Vpr-induced G2 arrest and apoptosis. PLoS One 9: e86840. 24466265 10.1371/journal.pone.0086840 PMC3899331

[8] Nonaka M, Hashimoto Y, Takeshima SN, Aida Y (2009) The human immunodeficiency virus type 1 Vpr protein and its carboxy-terminally truncated form induce apoptosis in tumor cells. Cancer Cell Int 9: 20. 19674438 10.1186/1475-2867-9-20 PMC2735735

[9] Nishizawa M, Kamata M, Katsumata R, Aida Y (2000) A carboxy-terminally truncated form of the human immunodeficiency virus type 1 Vpr protein induces apoptosis via G(1) cell cycle arrest. J Virol 74: 6058–6067. 10846089 10.1128/jvi.74.13.6058-6067.2000 PMC112104

[10] Aida Y, Matsuda G (2009) Role of Vpr in HIV-1 nuclear import: therapeutic implications. Curr HIV Res 7: 136–143. 19275582 10.2174/157016209787581418

[11] Nitahara-Kasahara Y, Kamata M, Yamamoto T, Zhang X, Miyamoto Y, et al (2007) Novel nuclear import of Vpr promoted by importin alpha is crucial for human immunodeficiency virus type 1 replication in macrophages. J Virol 81: 5284–5293. 17344301 10.1128/JVI.01928-06 PMC1900242

[12] Takeda E, Murakami T, Matsuda G, Murakami H, Zako T, et al (2011) Nuclear exportin receptor CAS regulates the NPI-1-mediated nuclear import of HIV-1 Vpr. PLoS One 6: e27815. 22110766 10.1371/journal.pone.0027815 PMC3218035

[13] Popov S, Rexach M, Zybarth G, Reiling N, Lee MA, et al (1998) Viral protein R regulates nuclear import of the HIV-1 pre-integration complex. Embo J 17: 909–917. 9463369 10.1093/emboj/17.4.909 PMC1170440

[14] Kamata M, Nitahara-Kasahara Y, Miyamoto Y, Yoneda Y, Aida Y (2005) Importin-alpha promotes passage through the nuclear pore complex of human immunodeficiency virus type 1 Vpr. J Virol 79: 3557–3564. 15731250 10.1128/JVI.79.6.3557-3564.2005 PMC1075686

[15] Felzien LK, Woffendin C, Hottiger MO, Subbramanian RA, Cohen EA, et al (1998) HIV transcriptional activation by the accessory protein, VPR, is mediated by the p300 co-activator. Proc Natl Acad Sci U S A 95: 5281–5286. 9560267 10.1073/pnas.95.9.5281 PMC20252

[16] Hashizume C, Kuramitsu M, Zhang X, Kurosawa T, Kamata M, et al (2007) Human immunodeficiency virus type 1 Vpr interacts with spliceosomal protein SAP145 to mediate cellular pre-mRNA splicing inhibition. Microbes Infect 9: 490–497. 17347016 10.1016/j.micinf.2007.01.013

[17] Kuramitsu M, Hashizume C, Yamamoto N, Azuma A, Kamata M, et al (2005) A novel role for Vpr of human immunodeficiency virus type 1 as a regulator of the splicing of cellular pre-mRNA. Microbes Infect 7: 1150–1160. 15908254 10.1016/j.micinf.2005.03.022

[18] Ayyavoo V, Mahalingam S, Rafaeli Y, Kudchodkar S, Chang D, et al (1997) HIV-1 viral protein R (Vpr) regulates viral replication and cellular proliferation in T cells and monocytoid cells in vitro. J Leukoc Biol 62: 93–99. 9225999 10.1002/jlb.62.1.93

[19] Hrecka K, Gierszewska M, Srivastava S, Kozaczkiewicz L, Swanson SK, et al (2007) Lentiviral Vpr usurps Cul4-DDB1[VprBP] E3 ubiquitin ligase to modulate cell cycle. Proc Natl Acad Sci U S A 104: 11778–11783. 17609381 10.1073/pnas.0702102104 PMC1906728

[20] Planelles V, Benichou S (2009) Vpr and its interactions with cellular proteins. Curr Top Microbiol Immunol 339: 177–200. 20012529 10.1007/978-3-642-02175-6_9

[21] Sherman MP, De Noronha CM, Williams SA, Greene WC (2002) Insights into the biology of HIV-1 viral protein R. DNA Cell Biol. 21: 679–688. 10.1089/104454902760330228 12396611

[22] Balotta C, Lusso P, Crowley R, Gallo RC, Franchini G (1993) Antisense phosphorothioate oligodeoxynucleotides targeted to the vpr gene inhibit human immunodeficiency virus type 1 replication in primary human macrophages. J Virol 67: 4409–4414. 8510229 10.1128/jvi.67.7.4409-4414.1993 PMC237816

[23] Connor RI, Chen BK, Choe S, Landau NR (1995) Vpr is required for efficient replication of human immunodeficiency virus type-1 in mononuclear phagocytes. Virology 206: 935–944. 7531918 10.1006/viro.1995.1016

[24] Dedera D, Hu W, Vander Heyden N, Ratner L (1989) Viral protein R of human immunodeficiency virus types 1 and 2 is dispensable for replication and cytopathogenicity in lymphoid cells. J Virol 63: 3205–3208. 2524599 10.1128/jvi.63.7.3205-3208.1989 PMC250884

[25] Lavallee C, Yao XJ, Ladha A, Gottlinger H, Haseltine WA, et al (1994) Requirement of the Pr55gag precursor for incorporation of the Vpr product into human immunodeficiency virus type 1 viral particles. J Virol 68: 1926–1934. 8107252 10.1128/jvi.68.3.1926-1934.1994 PMC236654

[26] Zhou T, Dang Y, Baker JJ, Zhou J, Zheng YH (2012) Evidence for Vpr-dependent HIV-1 replication in human CD4+ CEM.NKR T-cells. Retrovirology 9: 93. 23134572 10.1186/1742-4690-9-93 PMC3528630

[27] Schoggins JW, Rice CM (2011) Interferon-stimulated genes and their antiviral effector functions. Curr Opin Virol 1: 519–525. 22328912 10.1016/j.coviro.2011.10.008 PMC3274382

[28] Sadler AJ, Williams BR (2008) Interferon-inducible antiviral effectors. Nat Rev Immunol 8: 559–568. 18575461 10.1038/nri2314 PMC2522268

[29] Huang Y, Walstrom A, Zhang L, Zhao Y, Cui M, et al (2009) Type I interferons and interferon regulatory factors regulate TNF-related apoptosis-inducing ligand (TRAIL) in HIV-1-infected macrophages. PLoS One 4: e5397. 19404407 10.1371/journal.pone.0005397 PMC2672636

[30] Kohler JJ, Tuttle DL, Coberley CR, Sleasman JW, Goodenow MM (2003) Human immunodeficiency virus type 1 (HIV-1) induces activation of multiple STATs in CD4+ cells of lymphocyte or monocyte/macrophage lineages. J Leukoc Biol 73: 407–416. 12629155 10.1189/jlb.0702358

[31] Nasr N, Maddocks S, Turville SG, Harman AN, Woolger N, et al (2012) HIV-1 infection of human macrophages directly induces viperin which inhibits viral production. Blood 120: 778–788. 22677126 10.1182/blood-2012-01-407395

[32] Okumura A, Lu G, Pitha-Rowe I, Pitha PM (2006) Innate antiviral response targets HIV-1 release by the induction of ubiquitin-like protein ISG15. Proc Natl Acad Sci U S A 103: 1440–1445. 16434471 10.1073/pnas.0510518103 PMC1360585

[33] Pincetic A, Kuang Z, Seo EJ, Leis J (2010) The interferon-induced gene ISG15 blocks retrovirus release from cells late in the budding process. J Virol 84: 4725–4736. 20164219 10.1128/JVI.02478-09 PMC2863725

[34] Sirois M, Robitaille L, Allary R, Shah M, Woelk CH, et al (2011) TRAF6 and IRF7 control HIV replication in macrophages. PLoS One 6: e28125. 22140520 10.1371/journal.pone.0028125 PMC3225375

[35] Wang X, Chao W, Saini M, Potash MJ (2011) A common path to innate immunity to HIV-1 induced by Toll-like receptor ligands in primary human macrophages. PLoS One 6: e24193. 21904615 10.1371/journal.pone.0024193 PMC3164183

[36] Janket ML, Manickam P, Majumder B, Thotala D, Wagner M, et al (2004) Differential regulation of host cellular genes by HIV-1 viral protein R (Vpr): cDNA microarray analysis using isogenic virus. BiochemBiophys Res Commun 314(4): 1126–32. 10.1016/j.bbrc.2004.01.008 14751250

[37] Vázquez N, Greenwell-Wild T, Marinos NJ, Swaim WD, Nares S, et al (2005) Human immunodeficiency virus type 1-induced macrophage gene expression includes the p21 gene, a target for viral regulation. J Virol79(7): 4479–91. 10.1128/JVI.79.7.4479-4491.2005 PMC1061522 15767448

[38] Darnell JE Jr (1997) STATs and gene regulation. Science 277: 1630–1635. 9287210 10.1126/science.277.5332.1630

[39] Kane M, Yadav SS, Bitzegeio J, Kutluay SB, Zang T, et al (2013) MX2 is an interferon-induced inhibitor of HIV-1 infection. Nature 502: 563–566. 24121441 10.1038/nature12653 PMC3912734

[40] Honda K, Yanai H, Negishi H, Asagiri M, Sato M, et al (2005) IRF-7 is the master regulator of type-I interferon-dependent immune responses. Nature 434: 772–777. 15800576 10.1038/nature03464

[41] Marie I, Durbin JE, Levy DE (1998) Differential viral induction of distinct interferon-alpha genes by positive feedback through interferon regulatory factor-7. Embo J 17: 6660–6669. 9822609 10.1093/emboj/17.22.6660 PMC1171011

[42] Shuai K, Liu B (2003) Regulation of JAK-STAT signalling in the immune system. Nat Rev Immunol 3: 900–911. 14668806 10.1038/nri1226

[43] Takeda K, Akira S (2000) STAT family of transcription factors in cytokine-mediated biological responses. Cytokine Growth Factor Rev 11: 199–207. 10817963 10.1016/s1359-6101(00)00005-8

[44] Pilz A, Ramsauer K, Heidari H, Leitges M, Kovarik P, et al (2003) Phosphorylation of the Stat1 transactivating domain is required for the response to type I interferons. EMBO Rep 4: 368–373. 12671680 10.1038/sj.embor.embor802 PMC1319158

[45] Bovolenta C, Camorali L, Lorini AL, Ghezzi S, Vicenzi E, et al (1999) Constitutive activation of STATs upon in vivo human immunodeficiency virus infection. Blood 94: 4202–4209. 10590065

[46] Chaudhuri A, Yang B, Gendelman HE, Persidsky Y, Kanmogne GD (2008) STAT1 signaling modulates HIV-1-induced inflammatory responses and leukocyte transmigration across the blood-brain barrier. Blood 111: 2062–2072. 18003888 10.1182/blood-2007-05-091207 PMC2234049

[47] Ayyavoo V, Mahboubi A, Mahalingam S, Ramalingam R, Kudchodkar S, et al (1997) HIV-1 Vpr suppresses immune activation and apoptosis through regulation of nuclear factor kappa B. Nat Med. 3: 1117–1123. 10.1038/nm1097-1117 9334723

[48] Espert L, Degols G, Lin YL, Vincent T, Benkirane M, et al (2005) Interferon-induced exonuclease ISG20 exhibits an antiviral activity against human immunodeficiency virus type 1. J Gen Virol 86: 2221–2229. 16033969 10.1099/vir.0.81074-0

[49] Herbeuval JP, Boasso A, Grivel JC, Hardy AW, Anderson SA, et al (2005) TNF-related apoptosis-inducing ligand (TRAIL) in HIV-1-infected patients and its in vitro production by antigen-presenting cells. Blood 105: 2458–2464. 15585654 10.1182/blood-2004-08-3058

[50] Lum JJ, Pilon AA, Sanchez-Dardon J, Phenix BN, Kim JE, et al (2001) Induction of cell death in human immunodeficiency virus-infected macrophages and resting memory CD4 T cells by TRAIL/Apo2l. J Virol 75: 11128–11136. 11602752 10.1128/JVI.75.22.11128-11136.2001 PMC114692

[51] Huang Y, Erdmann N, Peng H, Herek S, Davis JS, et al (2006) TRAIL-mediated apoptosis in HIV-1-infected macrophages is dependent on the inhibition of Akt-1 phosphorylation. J Immunol 177: 2304–2313. 16887991 10.4049/jimmunol.177.4.2304 PMC1892167

[52] Laforge M, Campillo-Gimenez L, Monceaux V, Cumont MC, Hurtrel B, et al (2011) HIV/SIV infection primes monocytes and dendritic cells for apoptosis. PLoS Pathog 7: e1002087. 21731488 10.1371/journal.ppat.1002087 PMC3121878

[53] Foley JF, Yu CR, Solow R, Yacobucci M, Peden KW, et al (2005) Roles for CXC chemokine ligands 10 and 11 in recruiting CD4+ T cells to HIV-1-infected monocyte-derived macrophages, dendritic cells, and lymph nodes. J Immunol 174: 4892–4900. 15814716 10.4049/jimmunol.174.8.4892

[54] Poon B, Grovit-Ferbas K, Stewart SA, Chen IS (1998) Cell cycle arrest by Vpr in HIV-1 virions and insensitivity to antiretroviral agents. Science281(5374): 266–9. 10.1126/science.281.5374.266 9657723

[55] Fenard D, Houzet L, Bernard E, Tupin A, Brun S, et al (2009) Uracil DNA Glycosylase 2 negatively regulates HIV-1 LTR transcription. Nucleic Acids Res37(18): 6008–1. 10.1093/nar/gkp673 PMC2764447 19696076

[56] Langevin C, Maidou-Peindara P, Aas PA, Jacquot G, Otterlei M, et al (2009) Human immunodeficiency virus type 1 Vpr modulates cellular expression of UNG2 via a negative transcriptional effect. J Virol 83(19): 10256–63. 19625402 10.1128/JVI.02654-08 PMC2748015

[57] Nekorchuk MD, Sharifi HJ, Furuya AK, Jellinger R, de Noronha CM (2013) HIV relies on neddylation for ubiquitin ligase-mediated functions. Retrovirology10: 138. 10.1186/1742-4690-10-138 PMC3842660 24245672

[58] Laguette N, Brégnard C, Hue P, Basbous J, Yatim A, et al (2014) Premature activation of the SLX4 complex by Vpr promotes G2/M arrest and escape from innate immune sensing. Cell156(1–2): 134–45. 10.1016/j.cell.2013.12.011 24412650

[59] Adachi A, Gendelman HE, Koenig S, Folks T, Willey R, et al (1986) Production of acquired immunodeficiency syndrome-associated retrovirus in human and nonhuman cells transfected with an infectious molecular clone. J Virol 59: 284–291. 3016298 10.1128/jvi.59.2.284-291.1986 PMC253077

